# Comparison of Apparent Diffusion Coefficient and T2 Relaxation Time Variation Patterns in Assessment of Age and Disc Level Related Intervertebral Disc Changes

**DOI:** 10.1371/journal.pone.0069052

**Published:** 2013-07-26

**Authors:** Nan Wu, Hao Liu, Jun Chen, Luo Zhao, Wei Zuo, Yue Ming, Sen Liu, Jiaqi Liu, Xinlin Su, Baoxiang Gao, Zhiquan Tang, Guixing Qiu, Guolin Ma, Zhihong Wu

**Affiliations:** 1 Department of Orthopedics, Peking Union Medical College Hospital, Peking Union Medical College and Chinese Academy of Medical Sciences, Beijing, P.R. China; 2 Biology and Biomedical Sciences, Harvard Medical School, Boston, Massachusetts, United States of America; 3 Peking Union Medical College and Chinese Academy of Medical Sciences, Beijing, P.R. China; 4 Department of Radiology, China-Japan Friendship Hospital, Beijing, P.R. China; 5 Department of Radiology, The 305 Hospital of People’s Liberation Army, Beijing, P.R. China; Georgia Health Sciences University, United States of America

## Abstract

**Purpose:**

To compare the variation patterns of ADC and T2 values in different age and intervertebral disc (IVD) levels, thus to identify their sensitivities in assessing age and disc level related IVDs changes.

**Materials and Methods:**

The T2 and ADC values were recorded from 345 IVDs of 69 volunteers. Kendall's correlation analysis was used to identify the relationship between age and T2/ADC mean values respectively. The one-way analysis of variance (ANOVA) with post hoc analysis was then applied to test the differences of T2 and ADC values among different IVD levels and age groups, followed by linear regression analysis between age (<45 and >45 years) and T2/ADC mean values. This study was approved by the Ethics Committee of the Chinese Academy of Medical Sciences and the Peking Union Medical College Hospital.

**Results:**

Significant negative correlation was observed between age and T2/ADC mean values. The T2 and ADC values showed significant differences among IVD levels and among age groups except for T2 values in age group 1 (25–34 years) and group 2 (35–44 years), and for ADC values at L1–2 level. Both T2 and ADC values showed significant differences between young (age<45 years) and elderly group (age>45 years) at each IVD level. A linear relationship was observed between age and T2/ADC mean values in the elderly group as well as in the young group for the ADC mean values, while no such tendency was identified in the young group for the T2 mean values.

**Conclusions:**

ADC values may be a more sensitive parameter than T2 in assessing age and disc level related intervertebral disc changes.

## Introduction

Low back pain (LBP) is one of the most widespread and costly illnesses in modern society. In the United States, lifetime LBP prevalence is nearly 80%,costing more than $100 billion per year [1], [2]. Intervertebral disc degeneration (IDD), which results from structural alterations of the intervertebral disc (IVD), is the leading cause of LBP [3].

Compelling investigations into IDD have increased the demand of accurate and non-invasive diagnostic tools for IVD changes detection in the early stage of degeneration. Imaging techniques, such as magnetic resonance imaging (MRI) and computerized tomography (CT) have made great progress in this field. For example, T2-weighted MRI has been used in subjective visual grading systems for IDD [4]. On the other hand, quantitative imaging is being paid more attention mainly because of the relative objectivity in structure changes detection. It was reported that T2 relaxation timecould reflect the molecular environment of IVDs [5]. Recent studies have also demonstrated that the apparent diffusion coefficient (ADC), a quantitative parameter obtained from diffusion-weighted imaging (DWI), can sensitively detect microstructure changes of IVDs, and thus could be a potential tool for identifying the early changes of IDD [6].

However, application of T2 and ADC values in assessing IVD changes during IDD remains controversial. Kealey et al. [7]observed decreased ADC values in the degenerated IVDs compared with normal ones. Although the same results were reported by Kurunlahti et al. [8], the correlation between disc degeneration and diffusion patterns was not significant. Ludescher et al. [9] suggested that both T2 and ADC values were sensitive to detect changes of the IVD matrix in one day, while Niu et al. [10] reported that T2 quantitation but not ADC was more sensitive in detecting age-related disc changes and early stage of IDD. Although T2 signal intensity of the IVDs was significantly associated with the ADC values, no significant difference in ADC values among IVD levels was observed, resulting in disputes on the clinical applications of ADC [10].

Our study was designed to test the hypothesis that ADC value is a more sensitive indicator than T2 relaxation time does in the assessment of age and disc level related IVD changes. In this study, T2 and ADC values were acquired from 345 IVDs and then the variation patterns of these two parameters were compared in different age groups and anatomic levels.

## Materials and Methods

### 1. Subjects

Sixty-nine asymptomatic subjects (36 female, 33 male; mean age 45.1±10.6 years; age range 25–62 years) from one hundred and fifty two Chinese Northern Han population volunteers were enrolled. Inclusion criteria were as follow: no history of LBP or other spinal diseases (trauma, infection, inflammation, deformity, etc.); no history of heavy physical labor or heavy smoking; no contraindications for MRI scanning; no systemic disorders, such as diabetes, hypertension, hyperlipidemia. All participants signed the written informed consent, and underwent lumbar spine MRI scanning between January and April, 2011. This study was approved by the Ethics Committee of the Peking Union Medical College Hospital, Peking Union Medical College and Chinese Academy of Medical Sciences.

### 2. MRI Acquisition

All participants underwent MRI scans on a clinical 3.0-T scanner (Signa EXCITE, GE Medical Systems, Milwaukee, WI, USA). T2-weighted imaging was followed by diffusion-weighted imaging (DWI), with the following parameters: (1) sagittal and transverse T2-weighted imaging: fast spin-echo pulse sequence, repetition time (TR) = 4000 ms, echo time (TE) = 101.7 ms, section thickness = 4 mm, section gap = 1 mm, acquisition matrix = 384×256; (2) DWI: isotropic single-shot spin-echo echo-planar imaging sequence, TR = 4500 ms, TE = 72.9 ms, section thickness = 4 mm, section gap = 0 mm, acquisition matrix = 512×512. It took about six minutes to acquire the images in an individual subject.

### 3. Measurement of T2 and ADC Values

**Figure 1:**
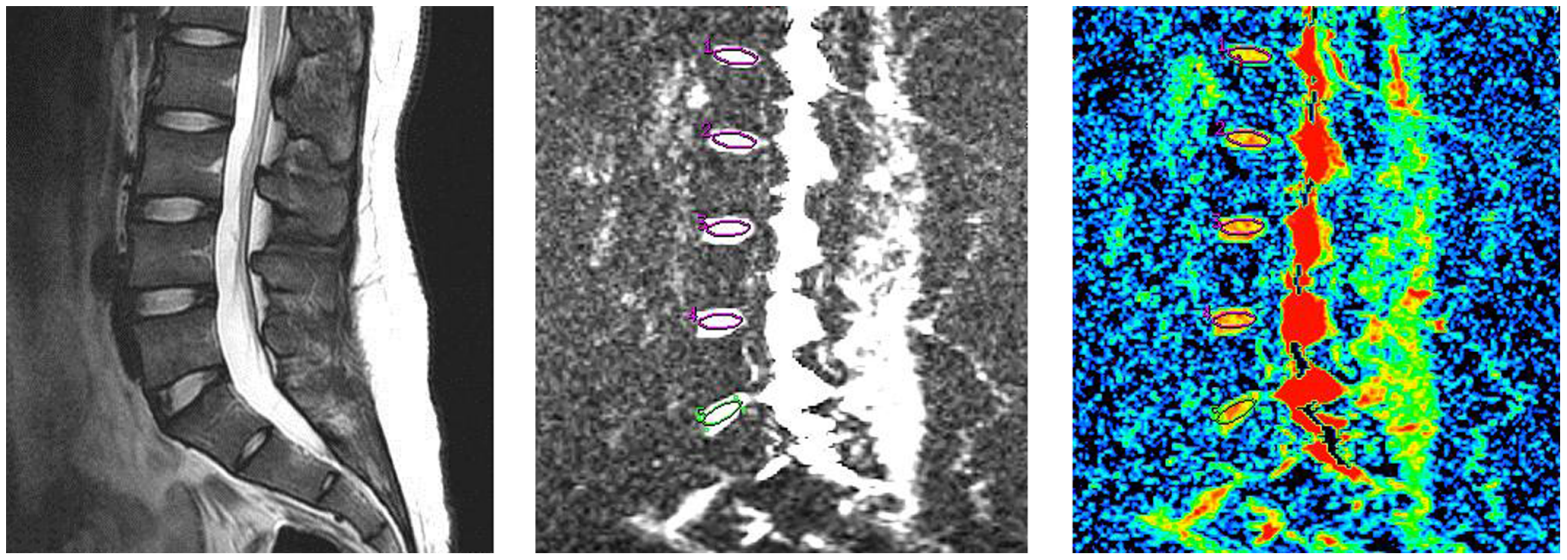
T2, DWI and ADC images of the lumbar intervertebral discs in a 26 years participant. The T2 intensity of the subject from L1–2 to L5-S1 were 92.6, 92.3, 95.1, 97.0, 95.8ms and the respective ADC values were 168, 178, 184, 182, 187×10^−5^ mm^2^/s.

The plane with the brightest IVDs of the three mid-sagittal T2-weighted images was chosen and an region of interest (ROI, 50 mm^2^) was placed centrally in the high signal area at each IVDs. An isotropic ADC map was calculated from three directional diffussion weighted images by using FuncTool Performance software (GE Medical Systems). The ADC values of each IVD, measured by using the signal intensity (I) attenuation according to the equation: ln[I(b1)/I(b0)] = -ADC×b, were obtained by drawing an elliptical ROI (90–110 mm^2^) on the ADC map **(** **Figure 1** **)**. All the measurements of T2 and ADC values were performed by a spine surgeon (with 3-year experience), a senior spine radiologist (with 7-year experience) and a senior spine surgeon (with 12-year experience) independently, and the mean values were then acquired for the following analysis.

### 4. Statistical Analysis

Kendall’s correlation analysis was firstly used to analyze relationship between age and the T2/ADC mean values respectively. All participants were then divided into four age groups (group 1∶25–34 years, group 2∶35–44 years, group 3∶45–54 years, group 4∶55–64 years). Analysis of variance (ANOVA) with post hoc test (Tukey’s honestly significant difference or Dunnett T3 test) was then performed to clarify the differences of T2 and ADC values among different IVD levels and age groups. Linear regression was finally used to identify the exact relationship between age and T2/ADC mean values in two age groups that were acquired based on the median age of all participants (young: <45 years; elderly: >45 years). The statistically significant threshold was set at *P*<0.05, and all the statistical analysis was performed using SPSS 15.0.1 (SPSS, Inc, Chicago, IL, USA).

## Results

A total of 345 IVDs images from 69 participants were collected. **Figure** **1** contains the representative T2, DW images and the corresponding ADC maps of a 26 years asymptomatic participant. The mean of the T2 and ADC values for all 345 IVDs were 81.8±19.1 ms and 161.8±25.2 ×10^−5^ mm^2^/s respectively. The detailed values of each age group and disc level were listed in **Table 1** and **Table 2**.

**Table 1:** One-way ANOVA for T2 intensity among age groups at each anatomical disc level.

Parameter	Age Group				*P* Value
	1 (25–34 years)	2 (35–44 years)	3 (45–54 years)	4 (55–64 years)	
T21	94.9±11.6	96.8±17.1	88.1±22.2	78.6±20.8	0.034
T22	101.3±20.9	100.9±19.9	79.2±25.0	67.9±26.8	<0.001
T23	107.0±17.3	96.7±20.5	78.3±27.9	57.4±21.9	<0.001
T24	99.6±24.1	83.5±17.7	71.5±28.1	56.5±21.9	<0.001
T25	103.4±31.8	81.4±35.8	65.0±20.2	56.3±29.8	<0.001
P Value	0.683#	0.073* #	0.035	0.035	

T2 intensity in different anatomical levels of every age group were showed as means±standard deviations (ms); T21, T22, T23, T24, T25 indicate the T2 intensity from L1–2 to L5-S1 IVD respectively;

* Brown-Forsythe test was used for ANOVA;

# *P*>0.05;

**Table 2:** One-way ANOVA for ADC values among age groups at each anatomical disc level.

Parameter	Age Group				*P* Value
	1 (25–34 years)	2 (35–44 years)	3(45–54 years)	4 (55–64 years)	
ADC1	170.6±12.9	170.2±14.5	165.9±19.4	158.8±20.1	0.179
ADC2	185.2±15.2	182.9±10.4	164.6±23.0	151.2±32.4	<0.001*
ADC3	189.9±10.5	179.4±10.5	161.4±24.8	141.6±26.0	<0.001
ADC4	181.7±14.1	167.1±17.3	151.4±28.0	137.6±25.7	<0.001*
ADC5	173.9±14.3	154.3±26.9	145.7±18.4	127.2±43.9	0.001*
P Value	0.002	<0.001	0.016	0.040	

ADC values at different anatomical levels of every age group were showed as means±standard deviations (×10^−5^ mm^2^/s); ADC1, ADC2, ADC3, ADC4, ADC5 indicate the ADC value from the L1–2 to L5-S1 IVD respectively;

* Brown-Forsythe test was used for ANOVA;

### 1. Variation Patterns of T2 and ADC Values in Different Age Groups

A significantly negative correlation was observed between age and the mean values of both T2 and ADC by using the Kendall’s correlation analysis (r = -0.481, *P*<0.001 for T2 and r = -0.533, *P*<0.001 for ADC) (**Table 3**).

**Table 3:** Kendall’s correlation analysis between parameters (mean value of T2 and ADC) and age.

Parameter	Correlation Coefficient (r)	*P* Value
Mean of T2 Intensity	−0.481	<0.001
Mean of ADC Value	−0.533	<0.001

T2 and ADC values significantly differed among four age groups at most anatomic levels except for the ADC value in the L1–2 IVD (F = 1.686, *P* = 0.179) (**Table 1** and **Table 2**). The source of these differences in paired subgroups was listed in **Table** 4 by using the post hoc analysis.

**Table 4:** Post hoc test in each anatomical disc level among age groups which are significantly different.

Subject	Age Group*	Age Group*	*P* Value	Subject	Age Group*	Age Group*	*P* Value
T22	25–34	45–54	0.008	ADC2#	25–34	45–54	0.016
		55–64	<0.001			55–64	0.005
	35–44	45–54	0.007		35–44	45–54	0.015
		55–64	<0.001			55–64	0.007
T23	25–34	45–54	0.001	ADC3	25–34	45–54	<0.001
		55–64	<0.001			55–64	<0.001
	35–44	45–54	0.018		35–44	45–54	0.009
		55–64	<0.001			55–64	<0.001
	45–54	55–64	0.006		45–54	55–64	0.004
T24	25–34	45–54	0.001	ADC4#	25–34	45–54	0.001
		55–64	<0.001			55–64	<0.001
	35–44	55–64	0.002		35–44	55–64	0.003
T25	25–34	35–44	0.043	ADC5#	25–34	45–5455–64	<0.001 0.003
		45–54	<0.001				
		55–64	<0.001				
	35–44	55–64	0.016				

* Unit: year;

# Dunnett T3 test is used; T21, T22, T23, T24, T25 indicate the T2 intensity from L1–2 to L5-S1 IVD respectively; ADC1, ADC2, ADC3, ADC4, ADC5 indicate the ADC value from L1–2 to L5-S1 IVD in every age group respectively.

Significant linear relationships were observed between age and the mean T2/ADC values in the elderly group (*df* = 38, *P*<0.001 for T2 and *df* = 38, *P* = 0.021 for ADC respectively); the same tendency was also found in the young group for the mean ADC value (*df* = 29, *P* = 0.011), but not for the mean T2 value (*df* = 29, *P* = 0.057) (**Table 5**). ANOVA among different disc levels showed significant differences in ADC values in both subgroups (F = 4.765, *df* = 4, *P*<0.001 for young group; F = 5.340, *df* = 4, *P*<0.001 for elderly group), and T2 values in the elderly group (F = 8.980, *df* = 4, *P*<0.001). However, no significant differences of T2 values were observed in the young group (F = 1.371, *df* = 4, *P* = 0.247) (data not shown).

**Table 5:** Linear regression analysis between parameters (mean value of T2 and ADC) and subject age (<45 years and >45 years).

Parameter	Age (years)	Coefficient	P Value
Mean of T2 Intensity	<45	−0.295	0.057*
	>45	−0.534	<0.001
Mean of ADC Value	<45	−0.368	0.011
	>45	−0.329	0.021

* *P*>0.05.

**Figure 2:**
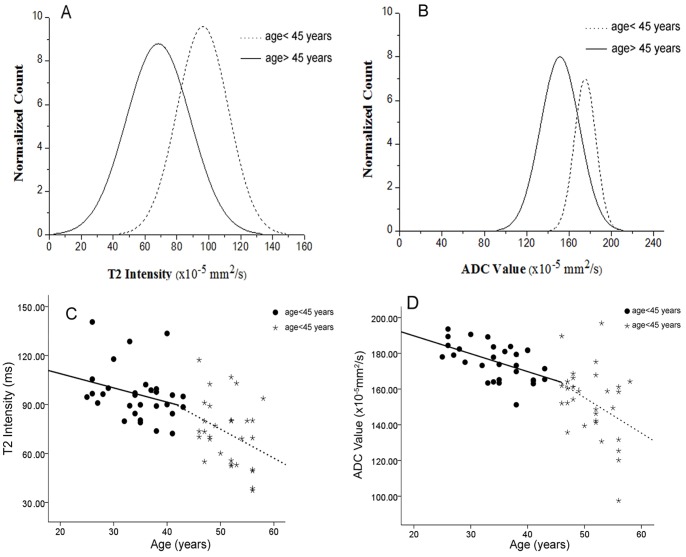
Normalized histograms and scatter plots of T2/ADC mean values in both young and elderly group. (A, B) Normalized histograms of T2 and ADC mean values for young (<45 years) and elderly (>45 years) groups. (C,D) Scatter plots of T2 intensity and ADC values versus subject age. The regression was to predict each value as a piecewise linear of age with breakpoint at age 45 years.

**Figure 2** **(A and B)** showed normalized histograms for the T2 and ADC values in both young and elderly groups, in which significantly lower values of T2/ADC in the elderly group than that of the young group (age <45) can be observed (T = 6.214, *df* = 67, *P*<0.001 for T2 and T = 6.826, *df* = 61.58, *P*<0.001 for ADC). **Figure 2** **(C and D)** showed the scatter plots of T2 and ADC values in both age groups.

### 2. Variation Patterns of T2 and ADC Values at Different IVD Levels

**Figure 3:**
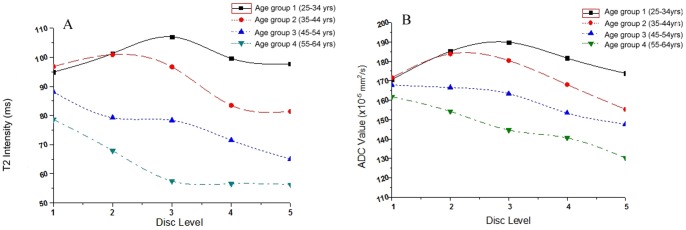
The line chart of T2/ADC mean values in each age group and IVD levels. In group 1 and 2, the T2 and ADC values increased gradually to their peak before steadily decreased. In group 3 and 4, both the two values declined continuously, no tendency of increase was seen.

T2 and ADC values significantly differed among anatomic levels in all age groups except for T2 values in group 1 (F = 0.574, *P* = 0.683) and group 2 (F = 2.288, *P* = 0.073) (**Table 1**). The sources of these differences were listed in **Table 6**. Significant differences of ADC values were found in group 1 and group 2, while no such difference existed for T2 values. Different from the continuously declined pattern in groups 3 and groups 4, T2 and ADC values increased gradually to their peak before steadily decreased in groups 1 and group 2 (**Figure 3**).

**Table 6:** Post hoc test which are significantly different (*P* value <0.05) among various disc levels in each age group.

T2 value				ADC value			
Age group*	IVD level	IVD level	*P* Value	Age group*	IVD level	IVD level	*P* Value
45–54	L1–2	L4–5	0.029	25–34	L1–2	L2–3	0.006
		L5-S1	0.003			L3–4	<0.001
55–64	L1–2	L3–4	0.014			L4–5	0.034
		L4–5	0.010		L2–3	L5-S1	0.030
		L5-S1	0.009		L3–4	L4–5	0.003
				35–44#	L2–3	L4–5	0.041
						L5-S1	0.008
					L3–4	L5-S1	0.022
				45–54	L1–2	L4–5	0.040
						L5-S1	0.004
					L2–3	L5-S1	0.008
					L3–4	L5-S1	0.026

* Unit: years; IVD, intervertebral disc;

# Dunnett T3 test was used;

## Discussion

The structural changes associated with IDD are characterized of impaired water diffusion in the degenerated IVDs [11]. Measurement of T2 values can reflect the water content of IVDs. However, changes in water content due to microstructural damage may occur before a significant loss of T2 signal intensity [12]. The ADC measurement gives an estimation of unbound water diffusion, and thus provides more detailed information about the microstructural changes [10]. Therefore, the ADC value may be a more promising parameter than T2 in the assessment of early vertebral changes.

In this study, values of T2 and ADC were measured in asymptomatic subjects, the variation patterns of these two parameters among different age and anatomical levels were compared. The mean of T2 and ADC values in all 345 IVDs were 81.8±19.1 ms and 161.8±25.2 ×10^−5^ mm^2^/s respectively, which were in the range of normal and degenerative values [13], [14], [15]. It was reported that degenerations could be found in IVDs of asymptomatic participants. In our study, 78 of all the 345 IVDs were degenerative according to Pfirrmann grading [4] (Grade III/IV/V) (data not shown), which may contribute to our findings.

### 1. ADC may be More Sensitive in Detection of Age-related IVD Changes than T2

The extracellular matrix of the IVD is mainly composed of collagen and proteoglycans [16], [17]. This collagen network plays a key role in maintaining the water content of IVDs, which was reported to gradually decrease with age [18], [19]. Several studies have assessed the respective advantages of T2 and ADC measurements in detection of age-related IDD. Kerttula et al. [14] assessed the suitability of ADC measurements in evaluating degeneration processes of the IVDs and compared with T2 relaxation time measurements. Their findings suggested that ADC measurements may more sensitive in detecting early degenerative changes of IVDs. However, other studies have found the opposite [5], [20], [21], [22]. In our study, both ADC and T2 mean values were negatively correlated with age. Significant differences in both T2 and ADC values were observed among age groups at all disc levels except L1–2, while no differences were found in the ADC values. These may be due to the more cephalic position of L1–2 bearing a lower stress. The above evidence suggests that ADC and T2 values may be useful for the detection of age-related IVDs degeneration. However, a significant linear relationship was found between age and ADC but not T2 values in the young group (<45 years), indicating that age related IVDs changes can be sensitively reflected by ADC values in the young participants; subsequent ANOVA analysis confirmed this effect. This result, which was consistent with what has been reported by Kerttula [14], suggests that ADC value is more sensitive than T2 intensity for detection of age-related changes. However, the existence of considerable overlap between ADC and T2 values of both normal and degenerative discs must be taken into consideration [10].

### 2. ADC Values may be More Sensitive in the Detection of Disc Level-related IVD Changes than T2 Values

Caudal IVDs, which bear a heavier mechanical stress, experience more degeneration than cephalic IVDs measured by both gross and histologic examinations [23], [24]. Several studies have reported lower ADC values of caudal IVDs than that of the cephalic ones [7], [8], [25]; However, Jaakko et al. [10] demonstrated no difference in ADC values for L3–4, L4–5 and L5-S1 IVDs. Similar results were also found for the T2 values. Our study revealed significant differences in the ADC values among disc levels in each age group, while no differences in T2 values were found in groups 1 and group 2. These findings suggest that ADC values may be more sensitive than T2 values in identifying degenerative changes related to anatomic levels, which is consistent with previous studies [6], [26].

### 3. Limitations

Several limitations could be found in the study. First, neither biochemical nor histologic assessment of IVDs was performed. However, the relationship between ADC values and biochemical changes of IVDs in IDD has been previously established in cadavers, and changes in the disc matrix (decreases in glycosaminoglycans or water content) of the nucleus pulposus have also been proven to correlate with ADC values. [6]. Second, because only asymptomatic subjects were included, the relationship between ADC and clinical symptoms such as LBP could not be determined. However, it was reported that the pain intensity of LBP patients can lead to the variations of ADC values [27], which will inevitably increase the confounding factors for the study. Third, our manual method for outlining the ROIs may result in subjectivity and bias, especially in the late stage degenerative IVDs with an unclear boundary between the nucleus pulposus and annular fibrosus [28]. To limit this effect, in our study, all the measurements of T2 and ADC values were performed by a spine surgeon, a senior spine radiologist and a senior spine surgeon independently, and then the mean values were acquired for the following analysis.

In spite of these limitations, our results suggest that ADC values may be superior to T2 values in detecting age and disc level related degenerative changes of IVDs during IDD.

### 4. Conclusion

In this study, T2 and ADC values were measured in asymptomatic subjects and the variation patterns of these two parameters were compared among different age groups and among different anatomic levels. Our results demonstrated that ADC values might be more sensitive than T2 values in assessing age and disc level related IVD changes. Further histologic studies are needed to validate the accuracy of the ADC values in the detection of clinical IDD before it is routinely used.
